# Pathophysiology, development, and mortality of major non-communicable diseases in metabolic dysfunction-associated steatotic liver disease: A comprehensive review

**DOI:** 10.7150/ijbs.117211

**Published:** 2025-09-03

**Authors:** Minjeong Kang, Jihun Song, Eun Seok Kang, Seohui Jang, Taeho Kwak, Yihyun Kim, Meng Sha, Hwamin Lee, Seogsong Jeong

**Affiliations:** 1 Department of Biomedical Informatics, Korea University College of Medicine, Seoul, Republic of Korea.; 2 Biomedical Research Center, Korea University Guro Hospital, Seoul, Republic of Korea.; 3 Department of Biomedical Science, CHA University, Pocheon, Republic of Korea.; 4 Department of Liver Surgery, Renji Hospital, School of Medicine, Shanghai Jiao Tong University, Shanghai, China.

## Abstract

Metabolic dysfunction-associated steatotic liver disease (MASLD), formerly known as nonalcoholic fatty liver disease and metabolic dysfunction-associated fatty liver disease, has emerged as a critical contributor to the global burden of non-communicable diseases (NCDs). Beyond its hepatic manifestations, MASLD is pathophysiologically connected to broader metabolic dysfunction, including insulin resistance, obesity, type 2 diabetes, and cardiovascular complications. This review critically examines the bidirectional associations between MASLD and major NCDs, including type 2 diabetes, cardiovascular disease, chronic respiratory disease, and cancer, focusing on shared mechanisms such as chronic inflammation, insulin resistance, oxidative stress, lipotoxicity, and epigenetic alterations. Furthermore, we explore disease-specific mortality patterns and mortality-related factors in MASLD patients across NCD domains. This review underscores the need for comprehensive and multidisciplinary strategies that address not only metabolic control but also systemic inflammation, immunometabolic dysregulation, and epigenetic alterations. Such integrative approaches are essential to mitigating the multisystem burden of MASLD and reducing mortality from its associated NCDs.

## Introduction

Non-communicable diseases (NCDs), including cardiovascular disease (CVD), cancer, type 2 diabetes (T2D), and chronic respiratory diseases, have emerged as major global health concerns 1. These diseases, linked by shared pathophysiological mechanisms such as metabolic dysfunction, chronic inflammation, and oxidative stress, are integral in understanding the connection between metabolic disorders and the onset of NCDs, particularly highlighting metabolic-associated steatosis and liver disease (MASLD) as a key factor contributing to the escalating global NCD burden 2.

Previously known as non-alcoholic fatty liver disease (NAFLD), MASLD, characterized by excessive hepatic fat accumulation without significant alcohol consumption, is increasingly seen not just as a liver-specific issue but as a condition deeply intertwined with systemic metabolic and inflammatory disturbances 3. Notably, MASLD has a strong association with major NCDs, including CVD, cancer, T2D, and chronic respiratory diseases, and it can contribute to multiple extrahepatic complications.

In addition to MASLD, steatotic liver diseases can be categorized based on alcohol consumption as metabolic-associated alcohol-related liver disease (MetALD) and alcohol-related liver disease (ALD). Given that alcohol consumption influences the incidence, risk, and mortality rates of major NCDs and related complications, considerable research has been conducted to examine the differences in end-stage liver disease and mortality risks among MASLD, MetALD, and ALD 4, 5.

However, the pathophysiological interactions between MASLD and individual NCDs, as well as the associated mortality risks, remain inadequately understood. Therefore, this review aims to explore the pathophysiological links between MASLD and NCDs such as CVD, cancer, T2D, and chronic respiratory diseases, and to assess their impact on cause-specific mortality (CVD-, cancer-, T2D-, and respiratory disease-related mortality) and overall mortality.

Specifically, this review addresses: 1 The shared pathophysiological mechanisms underpinning MASLD and major NCDs, 2 The interplay between MASLD progression and the development of NCDs, and 3 The impact of MASLD on cause-specific mortality, including CVD-, cancer-, T2D-, and chronic respiratory disease-related deaths, as well as all-cause mortality. By providing a detailed analysis of these topics, this review aims to clarify MASLD's central role in the context of NCDs and identify opportunities for integrated prevention and management strategies. By clarifying the central role of MASLD in the pathophysiology of NCDs, this review aims to contribute to the development of integrated prevention and management strategies.

### Shared pathophysiology between MASLD and NCDs

**Figure 1:**
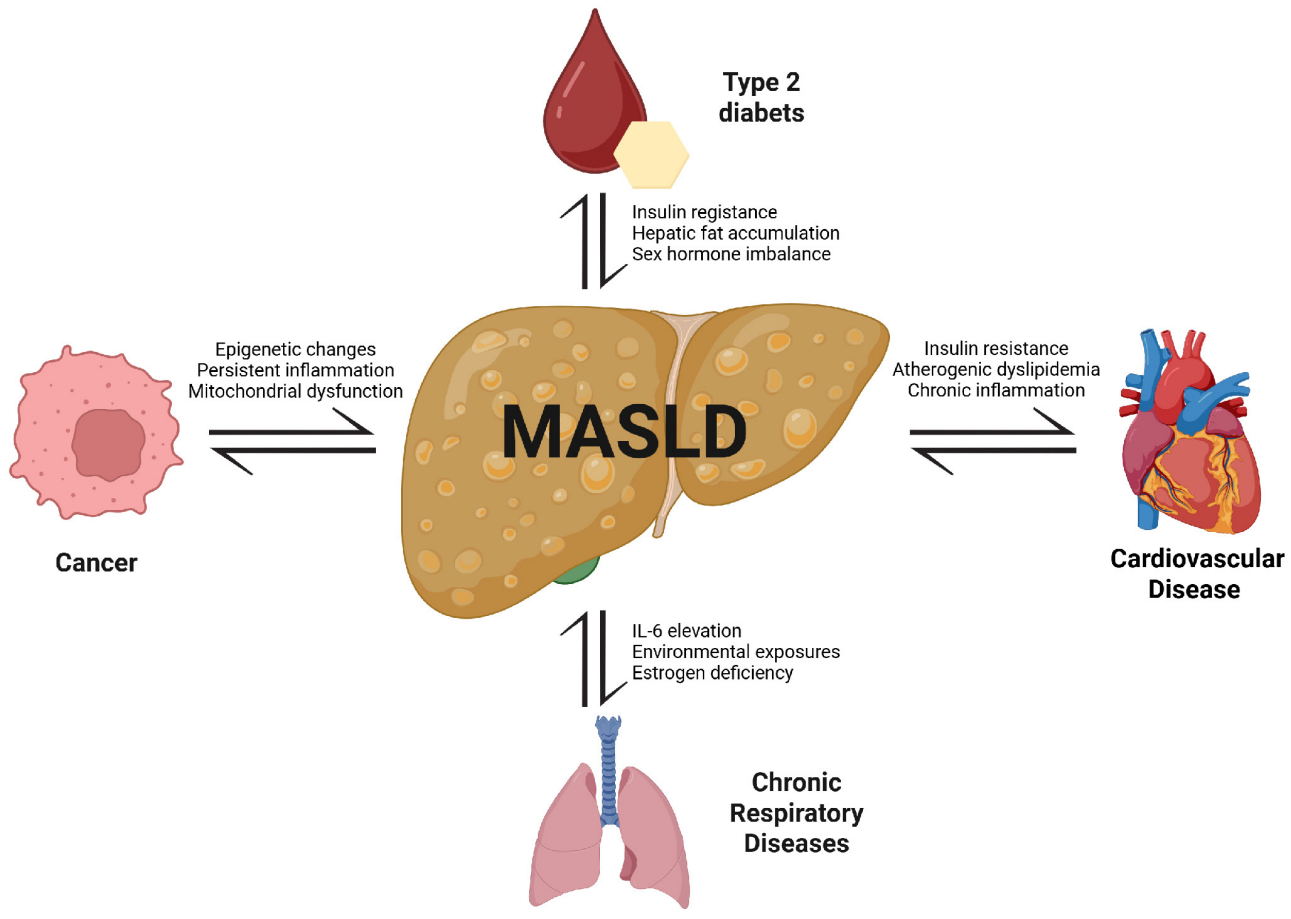
** Bidirectional associations between MASLD and major NCDs.** MASLD is centrally linked to the pathogenesis and progression of type 2 diabetes, cardiovascular diseases, chronic respiratory diseases, and cancer. Shared mechanisms include insulin resistance, chronic inflammation, dyslipidemia, hormonal imbalance, environmental exposures, and mitochondrial dysfunction. These pathways not only promote MASLD development but also exacerbate comorbid NCDs, creating a cycle of mutual reinforcement and increased mortality risk.

MASLD is a multifactorial disorder involving complex interactions among metabolic dysregulation, hepatic lipid accumulation, oxidative stress, and chronic inflammation. The disease process is initiated by sustained caloric excess and insulin resistance, which disrupt glucose and lipid homeostasis (Figure 1). Importantly, MASLD affects multiple organs through metabolic and inflammatory pathways shared with NCDs, with the gut-liver, liver-heart, and liver-pancreas axes serving as key inter-organ links that underscore its role as a central mediator of cardiometabolic and extrahepatic complications 6.

**Figure 2:**
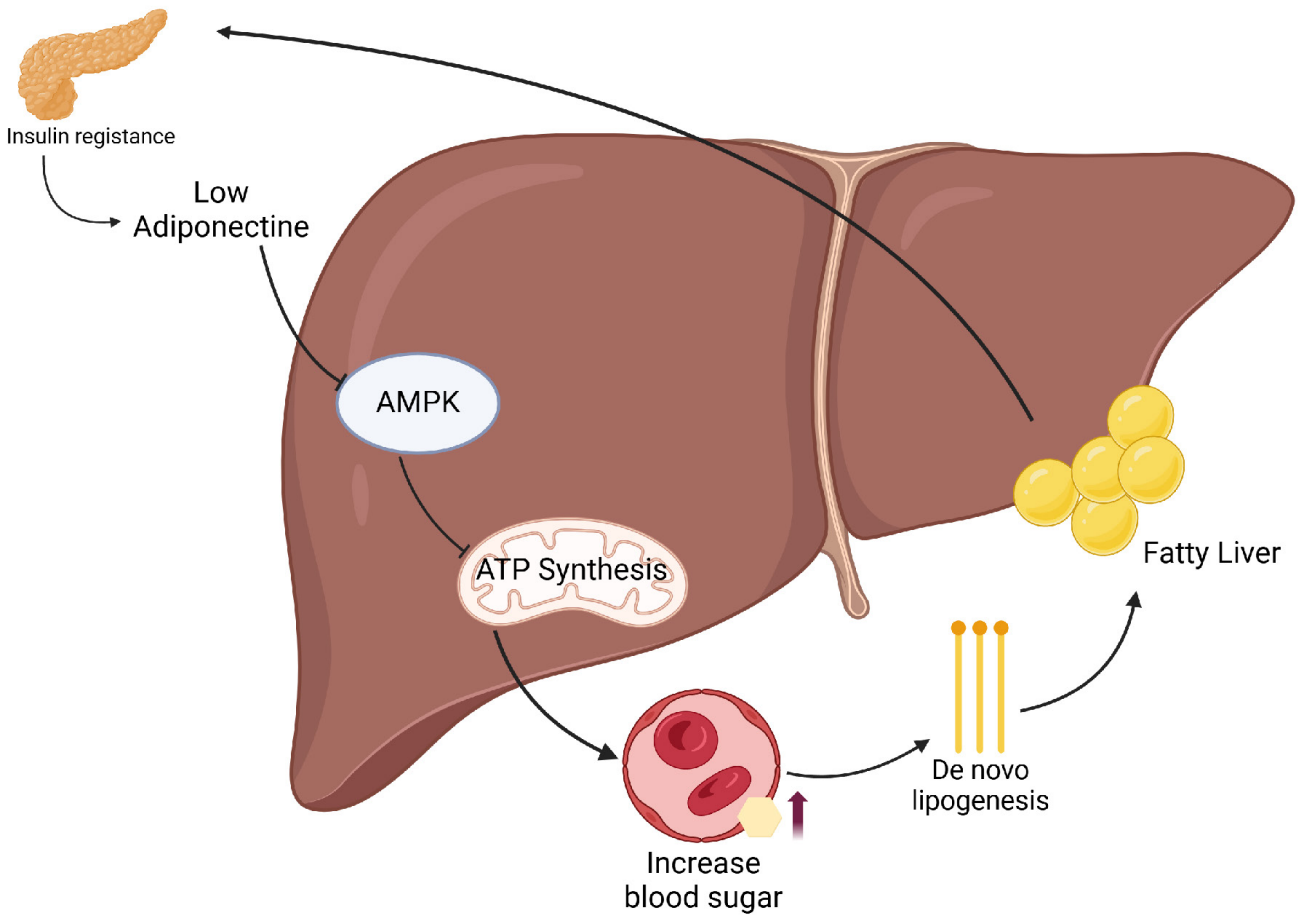
** Mechanistic pathway linking insulin resistance to MASLD via adiponectin-AMPK signaling.** Insulin resistance leads to decreased adiponectin levels, which suppresses AMPK activation in hepatocytes. This impairs ATP synthesis and enhances *de novo* lipogenesis, resulting in increased hepatic fat accumulation (fatty liver). Concurrently, hepatic insulin resistance disrupts glucose homeostasis, contributing to elevated blood glucose levels and further exacerbating metabolic dysfunction.

In insulin-resistant states, hepatic gluconeogenesis persists despite hyperinsulinemia. Concurrently, increased lipolysis in adipose tissue elevates free fatty acids (FFAs), which are transported to the liver. These FFAs promote *de novo* lipogenesis through the upregulation of sterol regulatory element-binding protein-1c (SREBP-1c) and carbohydrate-responsive element-binding protein (ChREBP) 7. Insulin resistance also reduces adiponectin secretion, further amplifying hepatic fat accumulation in a vicious cycle (Figure 2) 8. As hepatic lipid accumulation increases, mitochondrial dysfunction and endoplasmic reticulum (ER) stress impairs β-oxidation and increases the generation of reactive oxygen species (ROS), leading to lipid peroxidation and hepatocyte injury. The release of damage-associated molecular patterns (DAMPs) activates Kupffer cells and hepatic stellate cells, triggering NF-κB and NLRP3 inflammasome signaling, and including cytokines, such as TNF-α, IL-6, and IL-1β 9. Notably, chronic airway inflammation observed in diseases such as chronic obstructive pulmonary disease (COPD) and asthma has also been associated with elevated IL-6 levels 10, and emerging evidence suggests that MASLD-related activation of neutrophils and macrophages may similarly contribute to emphysema pathogenesis by releasing proteolytic enzymes that overwhelm alpha-1 antitrypsin (A1AT) regulation, a mechanism implicated in alveolar damage and disease progression in COPD 11-13. Furthermore, insulin resistance, a hallmark of MASLD, has been shown to double the risk of hepatocellular carcinoma (HCC) and increase its mortality by approximately 50% 14. While closely linked to T2D, insulin resistance also independently drives hepatic carcinogenesis through hyperinsulinemia and enhanced IGF-1 signaling, which activates the PI3K/AKT and MAPK pathways via insulin receptor substrate (IRS), promoting proliferation and inhibiting apoptosis 15-17. This oncogenic mechanism is observed even in the absence of overt T2D 18, although the coexistence of T2D in MASLD patients further amplifies the risk of HCC 19.

MASLD is also associated with dysregulated lipid metabolism. Patients often exhibit atherogenic dyslipidemia characterized by elevated small dense low-density lipoprotein (sdLDL), increased very low-density lipoprotein (VLDL) secretion, and decreased high-density lipoprotein (HDL) cholesterol 20. Epigenetic changes, such as aberrant DNA methylation of insulin signaling genes (e.g., IRS1, AKT2), histone modifications, and altered microRNAs (e.g., miR-122, miR-34a), further drive disease progression 21-23. Telomere shortening, p53 suppression, and loss of chromatin remodeling proteins (e.g., ARID1A) have also been identified in MASLD-associated HCC, linking genomic instability to carcinogenesis 24.

Environmental exposures, including endocrine-disrupting chemicals (EDCs) and air pollutants, such as PM2.5, contribute to hepatic steatosis and inflammation through oxidative stress, nuclear receptor interference, and gut-liver axis disruption 25, 26. The gut microbiota also influences MASLD pathophysiology via its metabolites, such as short-chain fatty acids (e.g., butyrate, acetate) that act as histone deacetylase (HDAC) inhibitors and modulate gene expression and immune responses. Microbial shifts affect the DNA methylation of genes involved in lipid metabolism and inflammation (e.g., APOA4, adiponectin, resistin), and modulate miRNA profiles related to hepatocyte apoptosis, fibrosis, and insulin sensitivity (e.g., miR-34a, miR-21, miR-582-3p) 27.

These mechanisms may vary by sex. Estrogen, SHBG, and ERα regulate lipid metabolism and immunity differently by sex 28. Epidemiological evidence shows that women with MASLD are at a higher risk of developing cirrhosis, while men have higher risks of hepatic decompensation, hepatocellular carcinoma (HCC; hazard ratio [HR], 2.59), cardiovascular disease (HR, 1.40), chronic kidney disease (HR, 1.16), and non-sex-specific cancers (HR, 1.32) 29. Similarly, asthma exhibits sex-dependent differences, with hormonal imbalance (e.g., excess estrogen in postmenopausal women) increasing disease burden 30. In men, testosterone enhances insulin sensitivity, and its deficiency has been linked to T2D onset in the context of MASLD 31. These hormonal imbalances may further exacerbate the metabolic burden of MASLD by promoting the development of NCDs.

### MASLD and the development of T2D

T2D is driven by a combination of insulin resistance, metabolic dysfunction, and chronic inflammation. Although these pathophysiological features are common to many NCDs, MASLD distinctly contributes to T2D progression by disrupting hepatic glucose metabolism and impairing pancreatic islet homeostasis 32.

The primary mechanism through which MASLD contributes to T2D development is hepatic insulin resistance 33. Accumulation of excess lipids in hepatocytes impairs insulin signaling, reducing the liver's responsiveness to insulin. As a result, gluconeogenesis proceeds unchecked, leading to increased hepatic glucose production and fasting hyperglycemia 34, 35. In response to insulin resistance, pancreatic β-cells temporarily compensate by increasing insulin secretion. However, prolonged exposure to lipotoxicity and glucotoxicity induces β-cell dysfunction and eventual insulin insufficiency, culminating in T2D onset 36.

Inflammatory cytokines elevated in MASLD, particularly IL-1β, contribute to β-cell toxicity and impaired insulin secretion, thereby compounding metabolic dysfunction and insulin resistance 37, 38. Beyond hepatic lipid accumulation, recent evidence highlights the pivotal role of the gut microbiota in modulating liver inflammation, oxidative stress, and systemic insulin sensitivity. Dysbiosis-related microbial components such as lipopolysaccharide (LPS), peptidoglycans, and flagellin can translocate from the gut to the liver, activate innate immune signaling, and exacerbate hepatic injury and insulin resistance. The absence of protective microbial metabolites such as indole-3-propionic acid (IPA) has been shown to worsen fibrosis and metabolic dysregulation, particularly in germ-free models or under environmental insults like smoking or high-fat diets 39.

MASLD-related epigenetic reprogramming, including methylation of insulin signaling genes (e.g., IRS1, AKT2) and altered miRNA expression, impairs glucose utilization and promotes mitochondrial dysfunction 40, 41. Accelerated epigenetic aging has also been observed, marked by DNA methylation changes and elevated inflammatory chemokines such as CXCL10, CXCL11, and enRAGE, all of which increase vulnerability to T2D 42, 43.

### MASLD and the development of CVD

Development of CVD is affected by various factors, such as air pollution, antibiotic use, body composition and weight, physical activity, cholecystectomy, and COPD 44-50. While MASLD is traditionally viewed as a hepatic manifestation of metabolic syndrome, hepatic steatosis itself with or without cardiometabolic risk factors has been identified as an independent predictor of cardiovascular morbidity and mortality 51-53, with the relative risk further increased in individuals with MetALD and ALD 54.

A key mechanism linking MASLD to CVD is vascular insulin resistance, which impairs endothelial function and promotes atherogenesis. In MASLD patients, lipotoxicity and chronic low-grade inflammation contribute to dysregulation of lipid metabolism, resulting in an atherogenic profile characterized by elevated small dense LDL, increased VLDL secretion, and reduced HDL cholesterol levels. These abnormalities accelerate plaque formation and heighten the risk of myocardial infarction, stroke, and heart failure 35. Under normal physiological conditions, insulin facilitates the degradation of apolipoprotein B (apoB), regulating plasma triglyceride levels. However, MASLD-induced insulin resistance disrupts this pathway, leading to increased triglyceride accumulation and altered lipoprotein composition, which further aggravate endothelial dysfunction and vascular inflammation 55.

**Figure 3:**
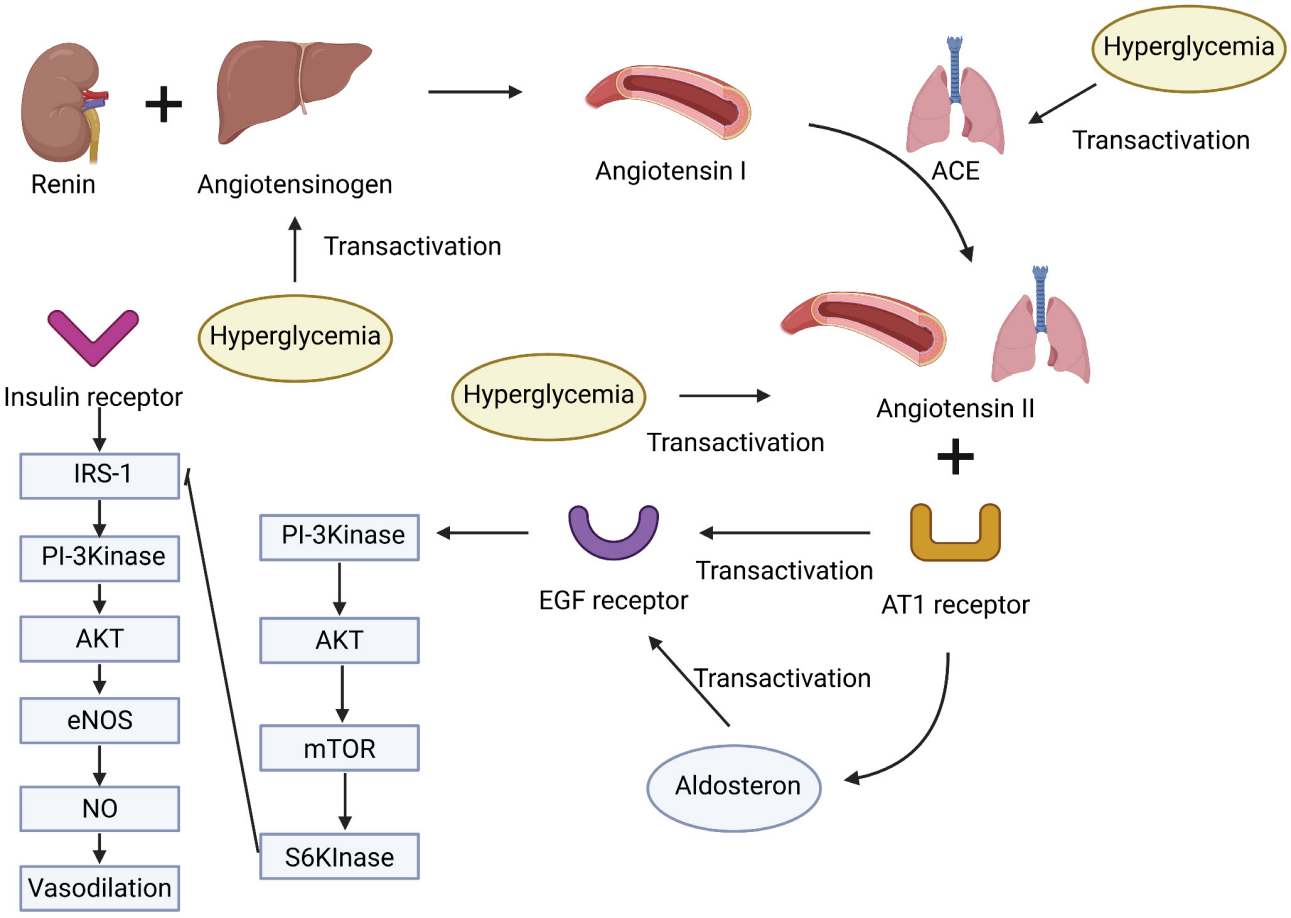
** Hyperglycemia and the renin-angiotensin-aldosterone system (RAAS) in MASLD.** Hyperglycemia promotes the upregulation of RAAS components, including angiotensinogen, ACE, angiotensin II, and the AT1 receptor. Angiotensin II stimulates aldosterone secretion and transactivation of the EGF receptor, while insulin signaling through the IRS-1/PI3K/AKT/eNOS pathway contributes to vasodilation. The interplay between these pathways may contribute to metabolic and vascular dysregulation in MASLD.

Overactivation of the renin-angiotensin-aldosterone system (RAAS) and heightened sympathetic nervous system activity, frequently observed in MASLD, contribute to increased vascular tone, sodium retention, and blood pressure elevation, thereby compounding cardiovascular risk 37. Hyperglycemia contributes to RAAS activation by increasing sodium-glucose reabsorption via SGLT2 and inducing oxidative stress, while insulin resistance enhances proximal sodium reabsorption, reduces sodium delivery to the macula densa, and stimulates renin release; these processes collectively activate the RAAS, upregulate components (such as angiotensinogen, ACE, and the AT1 receptor), and impair insulin signaling and nitric oxide bioavailability, thereby promoting endothelial dysfunction and elevating CVD risk in MASLD (Figure 3) 56-58. Moreover, persistent hyperglycemia in MASLD contributes to oxidative stress and impairs endothelial nitric oxide (NO) bioavailability, reducing vasodilatory capacity and promoting vascular stiffness 35. The intersection between MASLD and chronic kidney disease (CKD) also plays a role in CVD development. MASLD patients exhibit increased susceptibility to CKD, and the resultant decline in renal function exacerbates fluid imbalance and systemic inflammation, further elevating cardiovascular burden 59.

To date, the epigenetic mechanisms underlying the onset of CVD in patients with MASLD remain not fully understood. A study from Belgium found that gradual changes in DNA methylation is associated with progression of MASLD and epigenetic age acceleration 60. In MASLD, dysregulated DNA methylation disrupts phosphatidylcholine synthesis, which is critical for the maintenance of HDL and VLDL levels, thereby impairing metabolic homeostasis in a manner resembling atherogenic dyslipidemia, which is a well-established risk factor for CVD 61. Together, these epigenetic alterations may mechanistically link MASLD to increased CVD susceptibility.

### MASLD and the development of chronic respiratory diseases (COPD and asthma)

Chronic respiratory diseases such as COPD and asthma are increasingly recognized to share overlapping mechanisms with MASLD, including low-grade systemic inflammation, oxidative stress, and immunosenescence 62, 63. While traditionally regarded as distinct conditions, emerging evidence suggests that metabolic dysfunction driven by MASLD can negatively impact pulmonary physiology. Specifically, hepatic metabolic disturbances may contribute to systemic hypoxia and redox imbalance, ultimately compromising respiratory function and increasing susceptibility to pulmonary complications 63.

One mechanism through which MASLD affects COPD progression is by promoting pulmonary vascular dysfunction. Impaired NO bioavailability and elevated peripheral vascular resistance, frequently observed in MASLD, can disrupt pulmonary circulation and contribute to pulmonary hypertension. This hemodynamic alteration increases right ventricular workload and oxygen delivery deficits in COPD patients, compounding the burden of cardiopulmonary impairment 62.

MASLD also exacerbates chronic inflammation that fuels structural changes in the respiratory tract. Elevated levels of proinflammatory cytokines such as IL-6 and TNF-α are frequently observed in MASLD patients 64. These cytokines enhance airway remodeling and promote inflammatory cell recruitment, thereby accelerating the decline in lung function and increasing the frequency of COPD exacerbations 65. Beyond cytokine signaling, MASLD-related activation of innate immune cells, particularly neutrophils and macrophages, may contribute to emphysema pathogenesis by releasing proteolytic enzymes that disrupt A1AT regulation, a well-established mechanism in COPD 11, 12. While direct evidence is limited, this pathway offers a plausible immunometabolic link between MASLD and emphysematous lung changes. Recent studies also suggest that MASLD-related gut dysbiosis may contribute to pulmonary inflammation through the gut-lung axis, thereby exacerbating respiratory complications via immune and redox imbalance 39.

Another crucial impact of MASLD lies in its contribution to immune and metabolic dysfunction. By increasing susceptibility to chronic hypoxia and oxidative stress, MASLD accelerates immune aging and further deteriorates systemic metabolic regulation 63, 64. During acute COPD exacerbations, venous stasis and a hypercoagulable state increase the risk of pulmonary embolism (PE), and the accompanying hyperinsulinemia and vascular dysfunction in MASLD patients further exacerbate this risk 37, 66.

In asthma, MASLD is similarly implicated in amplifying airway inflammation. Due to heightened baseline inflammation, individuals with MASLD may experience more frequent and severe asthma exacerbations 67. Moreover, recent large-scale analyses have identified a significant association between MASLD and eosinophilic esophagitis (EoE), which is a chronic Th2-driven inflammatory disease that commonly develops in patients with asthma 68. Although the evidence is lacking, the increased risk of EoE may be associated with MASLD-driven asthma, which awaits future studies to confirm. Epigenetic modifications associated with MASLD, particularly aberrant DNA methylation, may further exacerbate airway inflammation by upregulating proinflammatory gene expression 69.

### MASLD and the development of cancer

Cancer development in patients with MASLD is driven by a combination of metabolic dysfunction, chronic inflammation, and epigenetic alterations, which collectively establish a tumor-promoting microenvironment 18. MASLD has been strongly associated with HCC, pancreatic cancer, colorectal cancer, and breast cancer, with hyperinsulinemia and activation of the IGF-1 signaling pathway playing central roles in tumor growth and metastasis 70. A pooled analysis of 18 cohort studies with conflicting results found that MASLD is associated with a higher risk of gastric, colorectal, thyroid, pancreatic, urinary system, biliary duct, skin, breast, and female genital cancers 71.

Chronic hepatic fat accumulation in MASLD leads to sustained hepatocellular injury, which impairs DNA repair mechanisms and increases genomic instability, thereby elevating the risk of tumorigenesis 72. Moreover, persistent oxidative stress and inflammation further contribute to hepatic tissue damage, fostering fibrosis and cirrhosis and ultimately raising the likelihood of HCC development 17. In MASLD, stressed hepatocytes release damage-associated molecular patterns, cytokines, and acute phase proteins that activate surrounding immune cells, contributing to a shift from hepatic immunotolerance to inflammation and driving progression toward fibrosis and HCC 73.

While HCC remains the most extensively studied cancer in MASLD, accumulating evidence suggests that systemic metabolic alterations in MASLD, such as hyperglycemia, insulin resistance, and inflammation, also contribute to the development of extrahepatic cancers 17. These abnormalities are particularly relevant in colorectal and pancreatic cancers, where chronic metabolic stress facilitates tumor proliferation and accelerates disease progression 70. In MASLD patients with coexisting T2D, insulin resistance within the colonic epithelium further promotes tumor growth and exacerbates cancer advancement 74. Notably, a recent cohort study suggested that improved glycemic regulation and weight management may reduce both liver and extrahepatic cancers 75. In addition, epigenetic modifications associated with MASLD also play a pivotal role in elevating cancer risk. Aberrant DNA methylation silences key tumor suppressor genes, including p16, p21, p27, and p53, thereby enhancing cellular proliferation, metastatic potential, and resistance to therapy factors that collectively contribute to poor cancer prognosis 76.

Recent evidence also highlights the influence of gut microbiota on tumor immunity. Dysbiosis-induced translocation of microbial products to the liver may trigger proinflammatory signaling and disrupt gut-liver immune homeostasis, potentially contributing to carcinogenesis, particularly in hepatocellular and colorectal cancers 39. In addition, metabolism-induced immune suppression in MASLD patients further facilitates tumor development 77, 78. Obesity, insulin resistance, and hepatic steatosis compromise immune surveillance, impairing the body's ability to eliminate malignant cells and increasing the risk of cancer initiation and metastasis 17.

Given the strong association between MASLD and cancer, early screening strategies targeting high-risk individuals are essential. Targeted anti-inflammatory therapies and metabolic regulation may serve to decelerate tumor progression and improve clinical outcomes in MASLD-related cancers 17, 78.

### Mortality in patients with MASLD and T2D

In the past, CVD was the leading cause of death in patients with T2D. However, improvements in blood glucose control, broader use of antihypertensive medications and statins, and lifestyle modifications have contributed to a reduction in CVD-related mortality 79. Nevertheless, when MASLD coexists with T2D, the overall mortality risk remains elevated, and cancer and CKD have emerged as leading causes of death 79.

Cancer plays a significant role in mortality among individuals with both MASLD and T2D. While hyperinsulinemia and systemic inflammation in T2D promote oncogenesis, MASLD exacerbates these mechanisms 80. Epidemiological studies indicate markedly increased rates of HCC, pancreatic, colorectal, and breast cancers in this population 80. Specifically, when T2D was diagnosed prior to MASLD, the risk of HCC increased by approximately 1.96-fold (HR 1.96; 95% CI: 1.69-2.27) and pancreatic cancer by 1.25-fold (HR 1.25; 95% CI: 1.06-1.48), whereas the risks of renal cancer (HR 1.12; 95% CI: 0.98-1.29), colorectal cancer (HR 0.92; 95% CI: 0.80-1.07), and breast cancer (HR 0.98; 95% CI: 0.88-1.09) were not significantly increased 41.

Furthermore, in individuals with T2D who were subsequently diagnosed with MASLD, the risk of HCC surged dramatically, while significant increases were also observed for pancreatic cancer (HR 1.78; 95% CI: 1.12-2.84), renal cancer (HR 2.01; 95% CI: 1.24-3.26), and breast cancer (HR 1.43; 95% CI: 1.09-1.88) 41. However, the elevated risk of colorectal cancer (HR 1.21; 95% CI: 0.81-1.81) was not statistically significant 41. MASLD-associated hepatic lipid overload and inflammatory signaling promote genomic instability, contributing to tumorigenesis in this high-risk group 81. Additionally, a reduction in 5-hydroxymethylcytosine (5hmC) levels in insulin signaling-related genes such as IRS1 and AKT2 has been identified in MASLD, suggesting potential early biomarkers for cancer risk 82, 83.

Biological aging acceleration, as reflected by DNA methylation patterns, has emerged as a mortality risk factor. Elevated levels of inflammatory mediators including CXCL10, CXCL11, and enRAGE, alongside CD8+ T cell activation, suggest immune dysregulation and increased vulnerability in MASLD-T2D comorbidity 40, 82.

When MASLD and T2D coexist, the likelihood of developing CKD rises substantially, which in turn aggravates cardiovascular mortality risk. As renal function declines, systemic inflammation and fluid dysregulation intensify, heightening the risk of heart failure. In advanced CKD, escalating insulin resistance further complicates metabolic control, contributing to a steep increase in cardiovascular-related deaths 59.

Importantly, *in vivo* studies have provided mechanistic evidence linking MASLD to elevated mortality in the context of T2D. ALOX15 was shown to exacerbate hepatic inflammation and oxidative stress in diabetic mice, thereby worsening insulin resistance and MASLD progression 84. Additionally, in a rat model of early NAFLD, insulin resistance was found to cause liver microcirculatory dysfunction, including impaired hepatic perfusion, increased portal pressure, and reduced sinusoidal endothelial fenestration. These microvascular alterations likely amplify hypoxia and hepatocellular injury, exacerbating inflammation and progression toward cirrhosis 85. This mechanistic link highlights the pathological role of insulin resistance not only in metabolic dysfunction but also in hepatic hemodynamic deterioration, contributing to the elevated mortality risk in patients with MASLD and T2D 85. These findings support the notion that MASLD contributes to mortality not only through comorbidity burden but also through direct metabolic and inflammatory insults.

Evolutionarily, severe insulin resistance once posed a survival and reproductive disadvantage. However, advancements in modern healthcare have allowed individuals with these metabolic traits to live longer and pass on such predispositions. Consequently, vulnerabilities that might have been diminished by natural selection are now more prevalent, contributing to the global rise in MASLD, T2D, and their associated mortality 86.

Accordingly, proactive prevention and tailored treatment strategies are crucial for individuals with comorbid MASLD and T2D. Rather than focusing solely on glycemic control, therapeutic efforts should prioritize systemic metabolic regulation and suppression of chronic inflammation. Incorporating lifestyle interventions, pharmacologic agents, and early biomarker-based risk stratification may enhance long-term survival prospects in this high-risk population.

### Mortality in patients with MASLD and CVD

CVD represents one of the most common causes of death in individuals with MASLD, primarily due to the convergence of metabolic dysregulation, atherosclerotic progression, and persistent inflammation 87. The link between MASLD and CVD is particularly robust. While hepatic fat accumulation has been identified as a feature of cardiometabolic syndrome and a predictor of cardiovascular mortality 88, the causal contribution of MASLD itself to cardiovascular death likely occurs through its impact on metabolic dysfunction and vascular injury.

Atherosclerotic progression is markedly accelerated in MASLD, translating into higher rates of cardiovascular death due to myocardial infarction, stroke, and heart failure 88. his is largely attributable to insulin resistance and lipid abnormalities, including elevated triglycerides and sdLDL, along with reduced HDL levels, which facilitate unstable plaque development and fatal vascular events 88.

Emerging *in vivo* studies provide mechanistic evidence linking MASLD with increased cardiovascular mortality. In a mouse model of MASLD, capillarization of liver sinusoidal endothelial cells (LSECs), characterized by loss of fenestrae and upregulation of capillarization markers such as CD31 and CD34, was shown to precede hepatic inflammation and was associated with elevated plasma LDL and triglyceride levels, supporting a causal role for LSEC dysfunction in systemic lipid dysregulation and cardiovascular disease progression 89. In this context, impaired hepatic clearance of chylomicron remnants due to LSEC capillarization may promote hyperlipidemia and atherosclerosis, key drivers of CVD-related mortality in MASLD 89.

Additionally, comorbid conditions such as obstructive sleep apnea syndrome (OSAS) and smoking may act as amplifiers of MASLD-induced cardiovascular damage. Periodic hypoxia in OSAS exacerbates hepatic steatosis via HIF2-α-mediated CD36 activation and increases systemic inflammation and insulin resistance in both mouse models and human studies, thereby compounding CVD risk 90. Smoking has similarly been shown to aggravate hepatic oxidative stress and gut-liver dysregulation in preclinical MASLD models, further promoting endothelial dysfunction and cardiometabolic complications 90.

Furthermore, MASLD may not only predispose individuals to CVD but also be worsened by CVD events. A recent *in vivo* study demonstrated that myocardial infarction (MI) in mice with MASH accelerates hepatic inflammation and fibrosis through Ly6Chi monocyte recruitment and elevated POSTN signaling, even though these mechanisms are typically associated with cardiac repair 91. These results suggest a bidirectional interaction, in which MASLD contributes to cardiovascular mortality while cardiac events simultaneously aggravate liver disease progression 91.

Persistent hypertension in MASLD patients, fueled by RAAS overactivity and sympathetic nervous system stimulation, contributes to vascular remodeling and arterial rigidity. These hemodynamic disturbances significantly raise the risk of cardiovascular mortality by intensifying end-organ damage and circulatory strain 37.

CKD acts as a compounding factor in the CVD mortality trajectory among MASLD patients. Declining renal function intensifies fluid overload and systemic inflammation, which together accelerate cardiovascular deterioration and contribute to higher fatality rates 59. To mitigate cardiovascular mortality in MASLD, prompt regulation of glycemic and lipid parameters is vital 88. Anti-inflammatory therapies also represent a crucial intervention, emphasizing the importance of integrated strategies aimed at curbing metabolic stress and vascular injury. Early screening and preventive interventions for cardiovascular disease in MASLD patients can significantly improve long-term survival outcomes.

### Mortality in patients with MASLD and chronic respiratory diseases (COPD and asthma)

The coexistence of MASLD with chronic respiratory diseases such as COPD and asthma significantly heightens mortality risk, largely due to the systemic complications induced by hepatic metabolic dysregulation. These include persistent inflammation, oxidative damage, and impaired immune responses, which collectively compromise pulmonary function and elevate the risk of fatal respiratory events 92.

Metabolic imbalances inherent to MASLD increase susceptibility to sustained hypoxic conditions and oxidative stress, both of which progressively undermine respiratory capacity and contribute to long-term pulmonary decline 64. Notably, mitochondrial oxidative phosphorylation (OXPHOS), enhanced by free fatty acid metabolism such as palmitate-induced β-oxidation, leads to the excessive production of ROS, including superoxide anions and hydrogen peroxide 93. These ROS exacerbate oxidative stress in MASLD, contributing to systemic insulin resistance and amplifying JNK signaling, a key modulator of inflammatory and apoptotic pathways 93. The elevated oxidative burden not only impairs hepatic insulin signaling but also damages pulmonary endothelial and epithelial cells, further deteriorating respiratory function in patients with coexisting MASLD and chronic respiratory conditions 93.

*In vivo* studies have begun to elucidate mechanistic links between MASLD and pulmonary pathology. A recent mouse study demonstrated that MASLD induced by a high-fat diet resulted in not only hepatic inflammation and fibrosis, but also significant structural damage in the lung, including increased pulmonary fibrosis and elevated expression of pro-inflammatory mediators 94. Treatment with quercetin, a flavonoid with anti-inflammatory properties, attenuated both hepatic and pulmonary injury, suggesting that MASLD-induced systemic inflammation directly contributes to respiratory disease progression 94.

In individuals with COPD, the presence of MASLD heightens the risk of pulmonary hypertension and cor pulmonale. This is largely driven by MASLD-induced reductions in NO bioavailability and elevated peripheral vascular resistance, both of which compromise pulmonary hemodynamics, restrict oxygen transport, and cumulatively increase cardiopulmonary strain, factors that significantly elevate long-term mortality risk in COPD patients 37.

Chronic low-grade inflammation driven by MASLD serves as a key accelerator of COPD progression. Elevated circulating levels of IL-6 and TNF-α, commonly observed in MASLD patients, contribute to worsening alveolar damage and persistent airway inflammation. Evidence further suggests that COPD patients with coexisting MASLD experience more frequent acute exacerbations, higher hospitalization rates, and increased risk of respiratory-related mortality 64.

The coexistence of metabolic dysfunction and immunosenescence in MASLD patients heightens vulnerability to oxidative stress and impairs host defense mechanisms. This immune compromise increases susceptibility to respiratory infections, thereby playing a significant role in the elevated mortality observed in COPD patients with MASLD 92. While MASLD may not act as a direct cause of death in chronic respiratory diseases, it likely exacerbates disease progression and outcomes through metabolic and inflammatory stress that compromises pulmonary physiology. Acute exacerbations of COPD are associated with an elevated risk of PE, which is further intensified in MASLD patients due to the presence of hyperinsulinemia and vascular dysfunction. This compounding effect may increase the vulnerability to acute cardiovascular events and related mortality in individuals with both conditions 95.

The presence of MASLD may contribute to increased asthma-related mortality by amplifying systemic inflammation and exacerbating airway vulnerability. As asthma is defined by persistent airway inflammation and heightened responsiveness to stimuli, the systemic inflammatory burden seen in MASLD may intensify disease severity and lead to more frequent and life-threatening exacerbations 96. Epigenetic alterations linked to MASLD, such as aberrant DNA methylation, may heighten inflammatory gene expression and exacerbate bronchial inflammation, thereby contributing to asthma-related mortality 97.

Severe asthma attacks and subsequent respiratory failure, often requiring mechanical ventilation, are the primary drivers of asthma-related mortality. In individuals with MASLD, hyperglycemia and systemic metabolic derangements impair immune surveillance, increasing vulnerability to infections and thereby elevating the risk of life-threatening asthma exacerbations 98. Thus, early screening and proactive interventions are essential for patients with coexisting MASLD and chronic respiratory diseases. Anti-inflammatory treatments and metabolic regulation strategies are crucial for reducing mortality and preserving lung function. Additionally, integrated management of respiratory function, vascular health, and immune status plays a vital role in improving the survival outcomes of MASLD patients.

### Mortality in patients with MASLD and cancer

Cancer-related mortality in MASLD patients has been on the rise, largely driven by factors that also underlie carcinogenesis, namely, metabolic imbalance, chronic inflammation, and epigenetic dysregulation 18, 99.

Rather than acting solely as an indirect contributor, MASLD may also exert direct effects that increase cancer mortality, as demonstrated by emerging *in vivo* evidence. A recent study revealed that CD8⁺ T cell exhaustion within the tumor microenvironment of MASLD-associated HCC leads to immune evasion and resistance to immune checkpoint blockade therapies. In particular, PD-1⁺CD8⁺ T cells exhibited reduced cytotoxicity, and their depletion accelerated tumor growth in MASLD mouse models, suggesting an intrinsic impairment in tumor surveillance driven by hepatic steatosis and inflammation 100. This finding underscores the functional link between MASLD and diminished anti-tumor immunity, which may worsen cancer prognosis 100.

In addition, suppression of ACMSD, a key modulator of NAD⁺ biosynthesis, has been shown to reduce γH2AX expression and improve mitochondrial function, thereby attenuating DNA damage, steatohepatitis, and hepatocarcinogenesis in MASLD models 101. Suppression of ACMSD has been shown to restore NAD⁺ levels and reduce γH2AX expression in MASLD models 101. Recent evidence further indicates that NAD⁺ depletion impairs DNA repair pathways, including RAD51 and γH2AX expression, providing mechanistic insight into how mitochondrial dysfunction and genomic instability contribute to MASLD-associated carcinogenesis 102.

Recent data also suggest that MASLD-associated hepatocarcinogenesis is exacerbated by genomic instability, including DNA repair failure, telomere shortening, and p53 pathway impairment, which have been observed in both clinical biopsies and *in vivo* mouse models 18, 72. MASLD is also known to heighten the risk of several malignancies, particularly HCC, pancreatic, colorectal, and breast cancers, with hyperinsulinemia and sustained IGF-1 pathway activation contributing not only to tumorigenesis but also to more aggressive tumor behavior and poorer prognostic outcomes 70, 103.

A major contributor to elevated cancer mortality in MASLD is the cumulative hepatocellular damage driven by steatosis and chronic oxidative stress. This ongoing injury compromises DNA repair capacity and promotes genomic instability, which not only increases the likelihood of tumor initiation but also accelerates HCC progression and reduces treatment efficacy 72. In advanced stages, cirrhosis, often a consequence of long-standing inflammation and fibrosis emerges as a pivotal determinant of poor survival in MASLD-associated liver cancer 104.

The increased cancer mortality associated with MASLD extends beyond hepatocellular carcinoma to include colorectal and pancreatic malignancies. In these cancers, metabolic disruptions, particularly chronic hyperglycemia, persistent inflammation, and insulin resistance, drive more aggressive tumor phenotypes and contribute to faster disease progression and reduced survival 97, 104. Among MASLD patients with comorbid T2D, insulin resistance within the colonic epithelium has been linked to enhanced tumor proliferation and metastatic potential, underscoring the compounded oncogenic risk in this population 105.

Beyond biological factors, socioeconomic disparities also significantly influence cancer mortality in MASLD. Patients with limited access to healthcare services are more likely to face delays in cancer diagnosis, leading to later-stage detection and diminished treatment outcomes. These systemic barriers contribute to the widening gap in survival rates among MASLD patients from underserved populations 106. This pattern is consistent with broader evidence indicating that poverty, inadequate education, and lack of insurance are associated with lower screening uptake, less access to standard-of-care therapies, and poorer overall survival, especially among racial and ethnic minorities 107. Even after adjusting for cancer stage, disparities in access to treatment, differences in physician recommendations, and challenges in patient, provider communication further contribute to unequal outcomes. Thus, the intersection of MASLD with socioeconomic inequality amplifies the burden of cancer mortality in vulnerable populations 107.

Given the elevated cancer risk in MASLD patients, expanding early detection efforts, especially for liver and colorectal cancers is vital for improving survival. Intensified screening protocols, coupled with targeted therapies aimed at reducing inflammation and restoring metabolic balance, may enhance treatment responsiveness and long-term outcomes in high-risk MASLD populations 108. Furthermore, addressing systemic barriers through public health initiatives, community outreach, and equitable access to screening and treatment resources should be prioritized to close the survival gap in MASLD-associated cancers 107.

In summary, early intervention and treatment strategies are essential to reduce cancer-related mortality in MASLD patients. Future research should not only focus on identifying specific therapeutic targets for MASLD-associated cancers but also on developing precision medicine and public health approaches that address both biological risk and social vulnerability 107.

## Conclusions and Future Directions

This review highlights the pivotal role of MASLD as a driver of NCDs and mortality, extending beyond hepatic pathology to systemic metabolic dysfunction. The findings underscore that MASLD not only exacerbates liver-related outcomes, such as cirrhosis and HCC, but also significantly increases the risk of CVD and T2D 109.

From a clinical perspective, the literatures emphasize the importance of addressing insulin resistance to mitigate the progression of MASLD and its related NCDs 110. In addition, recent studies have identified that comprehensive lifestyle interventions, including Mediterranean dietary patterns, caloric restriction, and structured physical activity (e.g., moderate aerobic exercise or high-intensity interval training), not only reduce hepatic fat and inflammation but also improve insulin sensitivity and cortisol regulation^111^, ^112^.

Future research should prioritize longitudinal studies, such as multi-center cohort studies or randomized controlled trials, which investigate the long-term risk of NCDs and mortality in MASLD patients. Stratified approaches targeting high-risk groups, such as individuals with advanced fibrosis or elevated FIB-4 scores, as well as stratification based on genetic and phenotypic characteristics, will also be essential for tailoring interventions and improving mortality outcomes 110, 113. By integrating MASLD into broader NCD prevention and management frameworks, healthcare providers may better address the high yet often overlooked burden of NCDs in patients with MASLD.
